# Changes in the Content of Phenolic Compounds and Biological Activity in Traditional Mexican Herbal Infusions with Different Drying Methods

**DOI:** 10.3390/molecules25071601

**Published:** 2020-03-31

**Authors:** Sandra N. Jimenez-Garcia, Moisés A. Vazquez-Cruz, Xóchitl S. Ramirez-Gomez, Vicente Beltran-Campos, Luis M. Contreras-Medina, Juan F. Garcia-Trejo, Ana A. Feregrino-Pérez

**Affiliations:** 1 División de Ciencias de la Salud e Ingeniería, Campus Celaya-Salvatierra, C.A. Enfermedades no transmisibles, Universidad de Guanajuato, Av. Ing. Javier Barros Sierra No. 201 Esq. Baja California, Ejido de Santa Maria del Refugio Celaya, Guanajuato, C.P. 38140, Mexico; sandra_neli_j@hotmail.com (S.N.J.-G.); xosofira2002@yahoo.com.mx (X.S.R.-G.); drvbeltranc@hotmail.com (V.B.-C.); 2 Departamento de Investigación y Desarrollo, KOPPERT MEXICO, Circuito el Marques Nte. 82, Parque industrial El Marqués, Santiago de Querétaro, C.P. 76246, Mexico; mvazquez@koppert.com.mx; 3 División de Estudios de Posgrado, C.A. Bioingeniería, Básica y Aplicada Facultad de Ingeniería, Universidad Autónoma de Querétaro, C.U. Cerro de las Campanas S/N, Colonia Las Campanas, Santiago de Querétaro, Querétaro, C.P. 76010, Mexico; miguel.contreras@uaq.mx (L.M.C.-M.); fernando.garcia@uaq.mx (J.F.G.-T.)

**Keywords:** phenolic compounds, biological activity, drying processes

## Abstract

Mexican spices are used in the supplementation of the human diet and as medicinal herbs for the particularly high amounts of compounds capable of deactivating free radicals. In addition, these spices can have beneficial effects on chronic, no-transmissible diseases such as type II diabetes and hypertension arterial. The objective of this study is to determine the content of phenolic compounds on the antioxidant activity and inhibitory enzymes of α-amylase, α-glucosidase and angiotensin-converting enzyme in melissa, peppermint, thyme and mint, which are subjected to microwave drying, conventional and freeze-drying to be used as alternative treatments. Spices were evaluated to determine total phenols, flavonoids, tannins, 2,2-Diphenyl-1-picrylhydrazyl (DPPH), (2,2′-azino-bis- (3-ethyl benzothiazolin-6-ammonium sulphonate) (ABTS) and Ferric Reducing/Antioxidant Power (FRAP), enzymatic activity. The investigation showed that conventional drying caused a decrease in antioxidant properties and inhibitory activity, in some species, while remained preserved in microwave drying and freeze-drying. The activity of polyphenol oxides and peroxidase decreases with high temperatures and these increase with the use of cold temperatures. This study aims to determine the extent of optimal drying required to preserve phenolic compounds, and the positive effect on antioxidant activity and enzymatic activity in in vitro models, which will produce benefits for the infusion processing industry and the pharmaceutical industry.

## 1. Introduction

The use of traditional medicinal herbs has become of daily use today. Most people prefer to use medicinal herbs that contain especially high amounts of compounds, capable of deactivating free radicals and generating health benefits, safety and efficacy, compared to pharmaceuticals. Some pharmaceutical products use plant extracts as raw material, in order to produce new medicines or as an active ingredient of existing medicines. On the other hand in developing countries, approximately 80% of the population depends on medicinal herbs for primary care, as well as in Oriental or Native American medicine [1]. One of the plants most commonly used as infusions of medicinal herbs in Mexico are herbs or spices that belong to the Lamiaceae family, as well as Melissa (*Melissa officinalis*), thyme (*Thymus vulgaris* L.), peppermint (*Mentha spicata*), and mint (*Mentha x piperita*). Its aerial parts, especially the leaves and flowers, are rich in secondary metabolites, such as tannins, rosmarinic acid, flavonoids, luteolin, apigenin, and phenolic compounds, essential oil, among other phytochemical compounds [2,3,4]. The benefits derived from the use of natural products, that are rich in bioactive substances, have promoted a growing interest in the pharmaceutical industries. Plants contain a wide variety of phytochemicals, antioxidants or bioactive molecules, which can counteract free radicals, and thus, retard the progress of many chronic diseases associated with oxidative stress and reactive oxygen species [5,6]. The consumption of infusions, or foods high in antioxidants, has been associated with beneficial effects against cancer, cardiovascular diseases, diabetes and other diseases associated with aging [7]. Therefore, antioxidants protect against free radicals derived from the glycation of non-enzymatic proteins, oxidative stress. Moreover, they accelerate in hyperglycemic conditions, causing complications in diabetic people, thus, humans have proposed as agents therapeutic to various herbs and spices. Furthermore, the antioxidant activity of herbs and spices, associated with type 2 diabetes, may be a primary factor in vascular diseases that diabetics often develop [8]. Due to the different effects of advanced protein glycation in diabetes and the pathologies caused by age, it has been proposed that the inhibition of glycation can prevent the progression of different pathologies of diabetic complications and delay aging [9,10,11]. Therefore, studies have focused on the characterization of phenolic and other phytochemical compounds with biological activity, which are supplied to human organisms as food components or as specific preventive pharmaceutical products, and can improve glucose metabolism, as well as the general health of diabetic patients [12,13]. Herbs and spices are generally used as infusions or as condiments in foods, are considered nutraceuticals concerning their nutritional, medicinal and therapeutic properties and, on the other hand, they also improve the taste of food, prolong shelf life by producing an antimicrobial activity [14,15]. The leaves of herbs and spices are processed before they are ready to be packaged. Drying the herb leaves is usually done to preserve the leaves, and thereby, increasing their shelf life [15,16]. Drying methods that are used, include air drying, sun drying, freeze-drying, oven drying, and microwave drying [17,18,19]. Drying removes water content by transferring the moisture content of the material to the surface, which prevents unwanted microbial growth, while increasing the stability of the phenolic compounds in the leaves [20]. It is a critical process, given it is the process is known to affect the antioxidant properties of the leaves due to thermal degradation and/or the action of oxidative enzymes [21]. Polyphenol oxidase is the oxidative enzyme degrading phenolic compounds, while peroxidase is related to phytochemical degradation in the presence of hydrogen peroxide, which plays a role in the degradation of phenolic compounds in plant leaves produced by different types of drying [22].

Research efforts have been currently oriented to the use of natural products, particularly plant foods or extracts in the management of chronic-degenerative diseases, such as the management of diabetes, high blood pressure, and other health-related challenges, as well as the conservation of these plant foods, which has deepened in the drying processes [19,23,24]. Therefore, the processes of drying herbs inhibit microbial growth and prevent certain biochemical changes but, at the same time, can lead to other alterations that affect the quality of the herb, such as changes in appearance and alterations of volatile compounds, reactions of oxidation or esterification reactions, and also, the increase in phytochemical compounds, such as eugenol, sesquiterpenes, and some phenolic compounds h [25]. Drying is necessary as a means to remove moisture and ensure that the product is enzymatically and microbiologically stable. However, previous research has also focused on the drying of various herbs associated with highly biologically active components [26]. Approximately 85% of these traditional herbs contain phytochemicals, which confers beneficial effects on health. The drying of aromatic herbs are considered a parameter for measuring quality, and water removal of the aromatic herbs are related to the drying methods. The production of new compounds can take place during drying. Any drying technology, innovative or traditional, that promotes an increase in bioactive potential would be of interest. The indicators commonly used to determine the quality of dried herbs, include sensory characteristics, structural properties and bioactive compounds, among others [27]. Microwave drying, conventional, freeze-drying are innovative, new and existing drying technologies, used in herbs or biomaterial processing, which help preserve biologically active components, and are an important aspect to be taken into account when applying a certain method of dried. Over the years, research have focused on studying the influence of drying on the chemical changes of various products. However, most previous studies have considered the effects of drying on antioxidant properties and content of essential oils [28]. Therefore, this document critically and comprehensively analyzes the effects of drying methods on the bioavailability of phenolic compound of various herbs. The objective of this paper is to review the influence of different drying methods on antioxidant activities, enzyme activity, and the contents of the phenolic compound of various herbs. Also, the advantages and limitations of the drying methods, used in this research, are analyzed in detail. Therefore, this paper aims to study the changes in the contents of the phenolic compounds of methanolic extracts to quantify different antioxidant compounds and bioactive compounds in Melissa, thyme, peppermint and mint in three drying methods, conventional, microwave, and freeze-drying. A study is also conducted to explore its health benefits and the effects of treatments and different types of spices.

## 2. Results

### 2.1. Quantification of Phenolic Compounds in Herbs and Spices

Data collected for phenolic compounds, which corresponded to the samples, were analyzed in the different drying treatments applied to the species. The identification of phytochemicals nowadays appears to be central in detecting the set of bioactive compounds in plants, as well as elucidating their antioxidant capacity and their bioactive capacity. Phenolic compounds are an efficient way to describe the interpretation of plant protective metabolites and the effect they can have on some chronic non transmissible diseases. With respect to the origin of the data shown in Table 1, the analysis of variance performed on the data for phenolic extraction showed a significant difference in the phenolic compounds by plant material in Melissa and thyme with a strong influence of the drying method by microwave and freeze-drying methods. Therefore, it can be observed that for 2 vegetative material analyzed such as Melissa and thyme, the greatest extraction of these compounds was achieved by the microwave drying with 0.5491, and 0.5343 mg GAE/g DW (F = 13.492; P = 0.000), respectively and freeze-drying method with 0.2897, and 0.3132 mg GAE/g DW (F = 15.407; P = 0.000) for Melissa, and Thyme, respectively. Peppermint showed significant difference in conventional drying with 0.1200 mg GAE/g DW (F = 27.695; P = 0.000). Peppermint concentrated the content of phenolic compounds up to 97% with the freeze-drying method and 95% with the microwave drying method, which indicates the appropriateness of these methods for the extraction of this functional compound. It is important to mention that the conventional drying method was the only method that caused a decrease in the concentration of phenolic compounds in peppermint, thyme, and mint by 16%, 5.0%, and 6.0%, respectively. The above gives indications of the susceptibility of these compounds to decomposition, employing a drying method where heat is used to carry out the drying process.

On the other hand, the results for the concentration of flavonoids, between drying methods, are shown in Table 1. Highly significant differences were observed between treatments. Again, microwave drying method and freeze-drying shows the best results for flavonoid extraction. In the case of thyme showed significant difference in the microwave drying method 0.93051mgRE/g DW (F = 125.032; P = 0.000) and for the mint showed significant difference in the microwave drying method and freeze-drying 0.87538 mg RE/g DW and 0.87356 mg RE/g DW (F = 200,684; P = 0.000) respectively. Microwave drying and freeze-drying methods allowed the flavonoid concentration to be increased by 88% in mint. However, it is important to note that freeze-drying is not recommended for the quantification of these compounds. Thyme showed a reduction in the flavonoid’s contents, compared to the treatment of fresh plant material, and the concentration was reduced to a 75%.

In the case of tannin extraction, significant differences were found between the drying methods evaluated. For most of the plant materials evaluated, freeze-drying was the better drying method. Thyme showed a concentration of 0.00224 mgCE/g DW (F = 501.007; P = 0.000). In peppermint, thyme, and mint, fresh plant material showed the highest concentrations. Unlike the phenolic and flavonoid compounds, none of the drying methods, used in the extraction of tannins, resulted an increase in the concentration of these compounds, and otherwise showed that the tannins are more sensitive to any drying method.

### 2.2. Analysis of the Different Antioxidant Capacity in Herbs and Spices

Data collected from the three different techniques were analyzed to determine antioxidant capacity, as shown in Table 2. The extraction by DPPH in the different drying methods showed highly significant differences. The freeze-drying method showed the highest concentration of DPPH for lemon balm and mint with 73.5%, and 77.5%, respectively, followed by the conventional drying method for thyme and mint 75.6%, and 76.4%, respectively. Otherwise, mint and peppermint showed a decrease DPPH concentration with respect to fresh plant material by 45%. The highest antioxidant capacity was shown in peppermint by the conventional drying method by 78% (F = 37.2; P = 0.000). On the other hand, the ABTS method illustrated the different plant materials showing significant differences in freeze-drying in three spices, such as, Melissa, peppermint and thyme. A decrease in antioxidant capacity was observed in peppermint by ABTS through the microwave drying method and conventional drying, compared to fresh plant material with 52%, and 40% respectively. This showed that peppermint could be more sensitive to the temperature changes used for the drying method. The highest antioxidant capacity by ABTS was shown by the conventional drying method in peppermint by 79% (F = 17.8; P = 0.000). Finally, for the FRAP method, the different drying methods showed a significant difference in the plant materials in the microwave drying method. A decrease in antioxidant capacity in Melissa and thyme was observed in the conventional drying method compared to fresh plant material with 88.5%, and 87%, respectively. This indicates that compounds with the ability to trap free radicals are more sensitive to heat and are, therefore, not determined as a drying method in this plant material. The plant materials with highest antioxidant capacity were the four spices (Melissa, peppermint, thyme and mint) in microwave drying method by 90% (F = 12.6; P = 0.000), 89% (F = 10.9; P = 0.000), 83% (F = 48.7; P = 0.000), and 88% (F = 11.7; P = 0.000) respectively.

### 2.3. Analysis of Bioactive Compounds of Herbs and Spices

Table 3 shows the plant materials in relation to the different drying method, in the inhibition of α-amylase, the samples show significant differences both in the plant material and in the drying method. In the factorial analysis, significant differences in the freeze-drying method were observed in the Melissa plant with 84% inhibition (F = 24.0; P = 0.000), increasing the inhibition capacity of α-amylase compared to fresh plant material in a 22%, On the other hand, peppermint did not show significant differences between the drying methods, but it did reflect a decrease in the percentage of inhibition in the microwave drying method with respect to the control by 65%. Likewise, in thyme and mint, there were no significant differences in the samples, and it behaves in the same way as peppermint. Thereby, the inhibition of α-glucosidase is affected by the drying method used in plant materials, and there is an inhibition of this enzyme in microwave drying methods in the four plant materials studied. As shown in Table 3 in Melissa, significant differences were not observed in microwave drying method in regard to fresh plant material. However, a decrease in α-glucosidase inhibition was shown in conventional drying by 44% compared to the fresh plant material. On the other hand, peppermint showed not greater inhibitory power on the α-glucosidase enzyme, by not showing a significant difference in the microwave drying method. Furthermore, freeze-drying suffers a decrease in this inhibitory activity. On the other hand, the conventional drying method showed to be more sensitive in regard to fresh plant material by 50% inhibition. Therefore, the heat used for the conventional drying method decreases phytochemical compounds with the ability to inhibit enzymes important in chronic degenerative diseases. Likewise, thyme and mint show no significant differences in the inhibition of the α-glucosidase enzyme. However, to finish the biological activities on the inhibition of the angiotensin-converting enzyme, the drying method that demonstrated the greatest effect was microwave drying method in Melissa, and mint with 55% (F = 43.1; P = 0.000), 72% (F = 180.8; P = 0.000), respectively. A decrease in the inhibition of angiotensin-converting enzyme in peppermint was also observed over the conventional and freeze-drying method by 46%, and 10%, respectively. In freeze-drying, g, the inhibition of angiotensin-converting enzyme was reduced by 6% with respect to fresh plant materials.

### 2.4. Analysis Comparative of Phytochemical Compounds in Different Plant Materials by Drying Method Using PCA.

**Figure 1:**
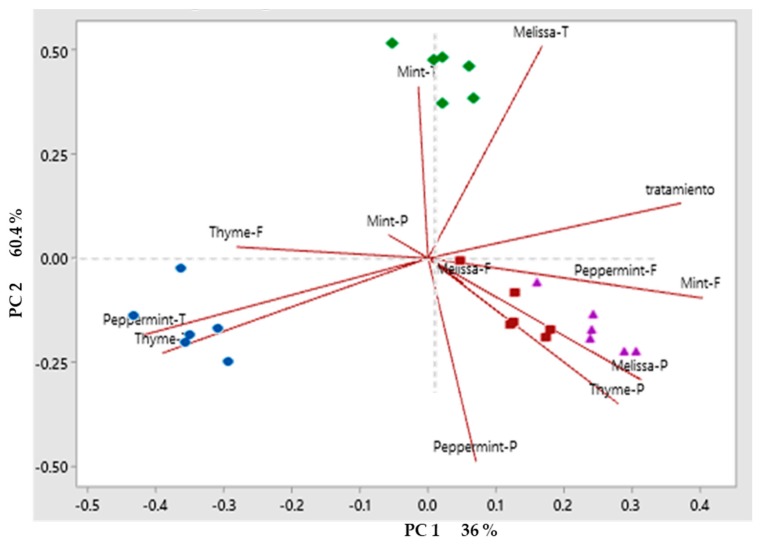
Principal component analysis (PCA) a) Phenolic compounds, flavonoids, tannins, by drying method and plant materials. [The different drying methods correspond to the Blue (Fresh plant), red (Microwave oven), green (Conventional) and violet (Freeze drying) icons] and [Acronyms per sample indicate P (Phenols), F (Flavonoids), and T (Tannins)] [Red line indicates the direction of the maximum variance of the data to orthogonal axes of each component].

**Figure 2:**
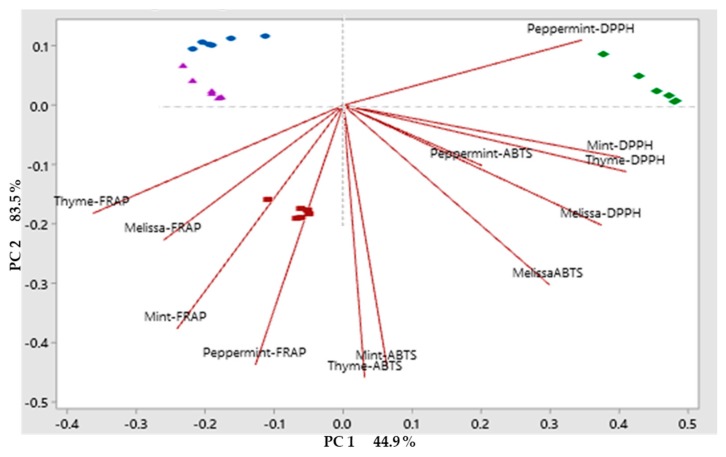
Principal component analysis (PCA) b) antioxidants capacity (ABTS, DPPH, FRAP), by drying method and plant material [the different drying methods correspond to the Blue (Fresh plant), red (Microwave oven), green (Conventional) and violet (Freeze drying) icons] [Red line indicates the direction of the maximum variance of the data to orthogonal axes of each component].

**Figure 3:**
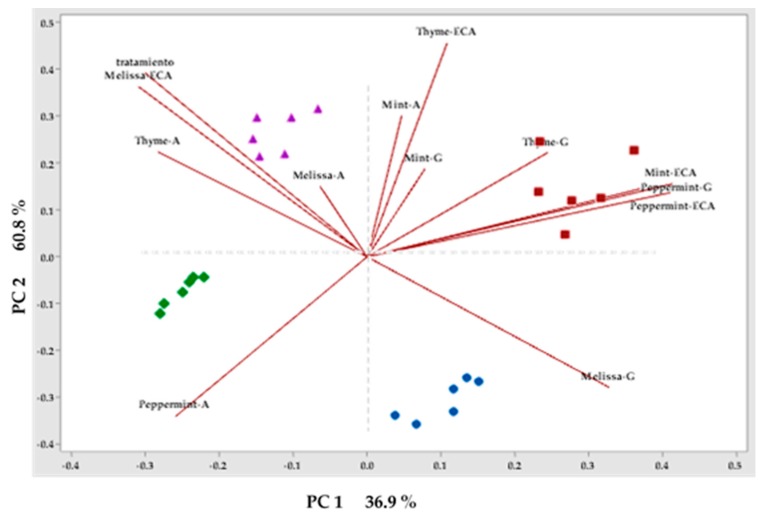
Principal component analysis (PCA) c) Biological activity (α-amylase, α-glucoside and ACE) by drying method and plant materials [the different drying methods correspond to the Blue (Fresh plant), red (Microwave oven), green (Conventional) and violet (Freeze drying) icons] and [Acronyms per sample indicate A (α-amylase), G (α-glucosidase), and ECA (Angiotensin converting enzyme)] [Red line indicates the direction of the maximum variance of the data to orthogonal axes of each component.].

**Figure 4:**
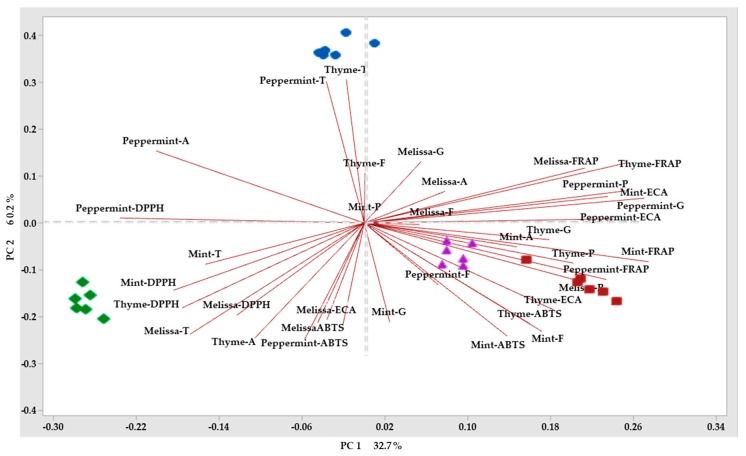
Principal component analysis (PCA) d) Phytochemical Compounds by drying method and plant materials [the different drying methods correspond to the Blue (Fresh plant), red (Microwave oven), green (Conventional) and violet (Freeze drying) icons] and [Acronyms per sample indicate P (Phenols), F (Flavonoids), T (Tannins), A (α-amylase), G (α-glucosidase), and ECA(Angiotensin converting enzyme)] [Red line indicates the direction of the maximum variance of the data to orthogonal axes of each component].

The analysis of the phytochemical compounds allowed us to identify the different compounds that were maintained after applying the drying method by plant material, the phytochemical compounds were the first analyzed by PCA, separating compounds by drying method. The analysis of the phenolic compounds is shown in (Figure 1). The two principal components (PC) described 78.8% of the variation among drying methods. PC1 describes 36% variation, whereas PC2 described 60.4%. Flavonoids in Melissa, peppermint, and mint, as well as the phenolic compounds in Melissa, peppermint and thyme described a positive variation in PC1 grouping in the drying methods with conventional drying method and freeze-drying. PC2 was described by Melissa and mint tannins grouped in conventional drying method. The drying methods that had of the greatest effect phenolic compounds were grouped into the microwave drying and freeze-drying methods. In the case of antioxidant capacity, the PCA showed that the first two principal components PC described 93.5% of the variation (Figure 2). In relation to antioxidant capacity, PC1 describes 44.9% of the variation, group the method by ABTS in the four plant materials studied, as well as the DPPH in Melissa, thyme, and mint. Whereas, PC2 describes 83.5% of the variation in the method by FRAP in the four plant materials. Grouping the three methods of quantification of antioxidant capacity in the microwave drying method. In the case of biological activity, the PCA showed that the first two principal compounds PC described 79.1% of the variation (Figure 3). Regarding the biological activity, PC1 describes 36.9% of the variation among treatments separating the enzymatic inhibition positively in Melissa by α-glucosidase and negatively in peppermint by α-amylase, PC2 described 60.8% of the variation in the biological activity grouping positively the biological activity in mint with the activity α-amylase, peppermint, thyme and mint with the activity α-glucosidase and ECA. The HCA (Hierarchical Cluster Analysis) groupings that grouped the greatest amount of biological activities were microwave drying method in a positive way and freeze-drying method in a negative way. On the other hand, in Figure 4, PCA was used to classify plant material according to their phytochemical composition affected by drying methods. HCA allowed the formation of clusters for the different drying methods, in order to compare their influence on phytochemical composition for the plant material. Variation for the 3 drying methods were explained in a 32.7% by the PC1. Whereas, PC2 allowed the explanation of 60.2% of the variation in composition for fresh plant material. PC1 separated drying methods in relation to the phytochemical compounds. However, it was not possible to see a clear separation, and the same occurred for PC2. Therefore, HCA appeared to be the most suitable tool in separating drying methods with respect to their influence on phytochemical composition for plant material. Therefore, as Figure 1, Figure 2, Figure 3 and Figure 4 show, there are large differences between the components in the case of fresh components and those after the different drying methods, which are hugely different in the technique used for this purpose.

## 3. Discussion

The different types of drying that are carried out in the spices have an influence on the content of bioactive compounds. Some may favor the detection of biologically active constituents that were not present in the raw material [5]. The results showed that antioxidant activity is favored by microwave drying and freeze-drying. The positive influence on the antioxidant potential of the extracts could be due, among other factors, to the stress caused by the plant structure. The factors that cause stress in plants to name a few are water deficit, soil composition, and temperature [4]. In this case, the high temperatures of the microwaves exert stress on the spices, generating great pressure on the cell walls, while during freeze-drying, the stress is due to the damage caused by the formation of ice crystals [29]. In both cases (freeze-drying and microwave drying), drying causes cell wall degradation facilitating the removal of water [18,21] and, in turn, the extraction of intracellular compounds, such as polyphenols. This would be a response to the high content of these Compounds in these drying methods. The polyphenols present in spices have positive effects on health, proving the effectiveness by in vitro tests of the elimination of free radicals (DPPH and ABTS) and the reduction of metal ions (FRAP) [30], in such a way that this could be an explanation of the antioxidant activities of spices. The biological activity of plants is shown to be useful for the treatment of various diseases, especially chronic non transmissible diseases such as diabetes. In the case of DMII, the inhibition of α-amylase has been considered as one of the important mechanisms involved in the control of diabetes [31,32]. The results obtained in the α-glucosidase test show that microwave-dried spices have increased hypoglycemic capacity in Melissa followed by microwave-dried mint. Melissa showed a significant reduction in blood glucose (65%; P < 0.05) in rats treated with the extract for 6 weeks [33] and in a study that included 62 patients who received capsules *M. Officinalis* (700 mg/d; *n* = 31) twice a day for 12 weeks, a significant reduction in HbA1c (hemoglobin A1c) (P = 0.002) was seen [8]. On the other hand, the inhibition of α-amylase has demonstrated its effectiveness of use of phenolic compounds for the inhibition of this enzyme in some studies carried out in diabetic rats with aqueous peppermint extract significant hypoglycemic properties were found [34]. On the other hand, a trial was conducted with male offspring of non-diabetic mothers and mothers with severe streptozotocin-induced diabetes. It was found that the offspring of diabetic mothers, treated with mint, showed significantly reduced glucose levels [9]. However, the content of polyphenols, could participate in the glucose metabolism pathways related to the absorption of glucose in the intestine, the secretion of insulin by the β-cells of the islets, the regulation of liver glucose production, insulin receptor activity in insulin-sensitive tissues and glucose uptake, and regulation of intrahepatic glucose production [10]. Numerous studies have shown that hypoglycemic properties related to phytochemical content, 26 such compounds, polyphenols, show a fasting and postprandial blood glucose response have been investigated in animal models and in human studies [35]. It is also possible to consider the synergistic effect of polyphenolic compounds, which may have a positive effect on blood glucose [10]. On the other hand, another of the important chronic noncommunicable diseases is Arterial Hypertension (HT). An in vitro test of ACE inhibition was performed, showing in our study significant changes in vitro tests we can show that spices can have a transcendental role in the treatment of HT. In the review of the literature, no studies were carried out on the spices used in a study with rats and Melissa aqueous extract at different doses (50, 100 and 200 mg/kg/day) where no significant effect on pressure arterial was observed [13]. It is evident that the phytochemicals that we found in spices have an effect against noncommunicable diseases and the drying methods that were applied in this investigation help us to corroborate the conservation of these phytochemicals with the use of the proper drying method. Other hand, according to Śledź, et al. [36], the loss of polyphenolic compounds in oregano ranged between 71.6 and 78.4%, depending on the microwave power applied, and increased when the microwave power was reduced. This can be related to the results of the samples analyzed since using this method of drying had losses of some phytochemical compounds, so it would now have to work with less power or less drying intervals. In some cases, as in mint, some authors also show data on the increase in polyphenols, attributing it to the breakdown of cellular components or ascorbic acid concentrations. Therefore, we must be careful with the techniques used in quantification of phenolic compounds by Folin-Ciocalteu’s [16]. Some authors classify Melissa and Thyme as woody herbs, so this type of tissue acts as a barrier to the release of phenolic compounds in the extracts and therefore results in a lower antioxidant capacity. On the other hand, lyophilized samples were dried at a very low temperature (−54 °C), and degrading enzymes could still be activated when they come in contact with moisture from any source, for example, probably from the air. In thyme, the difference in antioxidant capacity and total phenols between conventional drying and freeze-drying was greater. This is probably due to the fact that during conventional drying the leaves of the herbs are heated (70 °C) and their intercellular spaces collapse, releasing more phenolic compounds [19]. The results obtained from the ABTS, DPPH, FRAP trials showed a high degree of correlation. The highest correlation between the content of phenolic compounds and DPPH values was observed (Figure 4), as expected, as both tests followed the same principle of electron transfer based on the antioxidant effects against oxygen free radicals.

## 4. Materials and Methods

### 4.1. Sample Preparation

The herbs or spices (Melissa, thyme, peppermint, and mint) analyzed for this study were grown free of fertilizers and pesticides. The selection of these spices was made considering their good phytosanitary state (the spices should not have mold or any visible pest) and in a fresh state; the leaves and stems of the plants will be taken as part of the sample. In all cases, 1kg of flowering stems were collected. Samples were collected in a full flowering period, in the experimental de la Autonomous University of Queretaro campus Amazcala. The collected samples were divided into four parts and different drying methods were applied. Once selected, they were freeze-dried, conventionally dried and microwaved, as well as subsequently stored in closed containers, thereby eliminating oxygen with N_2_ gas to eliminate O_2_ in the headspace and stored in fresh and dry spaces for analysis. On the other hand, fresh samples were only frozen with liquid nitrogen and kept frozen at −18 °C for further analysis. The preparation of the sample for the analysis of phytochemical compounds the extraction will be carried out according to the methodology described by Cardador [37], 25 mg of dry sample and 200 mg of the fresh sample will be placed according to the case of study and added 2.5 mL of methanol to each sample, realizing samples in triplicate. They were kept free of light and stirred for 24 h. Centrifuging at 5000 rpm/10 min/4 °C, the pellet formed in the bottom was removed, leaving the supernatant. The sample is presented per g of dry sample.

### 4.2. Drying Methods Used in Herbs and Spices

Three drying methods were performed on each of the herbs and spices. Conventional drying will use a food dehydrator (Hamilton Beach 32100A, NC, USA) a temperature of 58 °C for a period of 25 h [38]. The microwave drying process will undergo a sample of 30 Ghz/10 s drying in 10 consecutive intervals in a microwave (Mabe HMM70SW, Santiago, Chile) [39]. For freeze-drying, it was performed in a freeze dryer (Labconco FreeZone 4.5L model 230V, Kansas City, USA) with a temperature of −50 °C with a vacuum system of 0.01 atm [40]. Table 4 shows the data of the parameters of each drying method.

### 4.3. Determination of Total Phenolic Compounds

The total phenol content was determined using the Folin-Ciocalteu spectrophotometric method [6] modified for use in 96-well microplate. In quantification, an aliquot of the methanolic extract (4 µL) mixed, 250 µL of the Folin-Ciocalteu reagent (1N), add 1250 µL of Na2CO3 (20%) were mixed and allowed to stand in the dark for 2 h at room temperature. Subsequently, the absorbance ismeasured at 760 nm in a spectrophotometer (MULTISKAN GO, Thermo Fisher Scientific, Finland). The results are expressed as mg equivalent of gallic acid/g DW.

### 4.4. Determination of Total Flavonoids

The spectrophotometric method used for the quantification of total flavonoids in methanolic extracts was determined by Oomah, et al. [41]. 50 μL of the methanolic extract was mixed with 180 μL of methanol and 20 μL of 1% 2-aminoethyldi-phenylborate solution in a 96-well plate. The experiments were performed in triplicate. The absorbance will be measure at 404 nm. Expressing the results in rutine hydrate mg equivalent/g DW.

### 4.5. Determination of Total Tannins

The quantification of total condensed tannins was performed following the procedure described by Feregrino–Pérez, et al. [42], modified for 96-well microplate. 50 μL of the methanolic extract was placed in the 96-well microplate with 200 μL of 1:1 solution (*v*/*v*) (1% vanillin - HCl 8. The number of condensed tannins in plate reader was quantified (MULTISKAN GO, Thermo Fisher Scientific, Finland) at 492 nm. Expressed in mg equivalent of (+) catechin/g DW and the samples were analyzed in triplicate.

### 4.6. Determination of Antioxidant Capacity

#### 4.6.1. DPPH Method Antioxidant Capacity

The quantification of DPPH antioxidant capacity (2,2-Diphenyl-1-picrylhydrazyl) was carried out as described by Zenil, et al. [43]. A total of 20 μL of the methanolic extract was mixed with 200 μL DPPH in a 96-well plate. The experiments were performed in triplicate. It was measured at an absorbance of 520 nm at a time of 30 min. The results were expressed in mg equivalent of trolox/g DW or in% radical inhibition ${\%}^{inhibición}=\frac{\begin{array}{c} \left(Abs.Inicial\right)-\left(Abs.Final\right) \end{array}}{Abs.Inicial}*100$.

#### 4.6.2. ABTS Method Antioxidant Capacity

The spectrophotometric method for the quantification of antioxidant capacity by ABTS (2,2′-azino-bis- (3-ethyl benzothiazolin-6-ammonium sulphonate) was performed following the Re method [44]. For quantification, 230 μL of ABTS with 20 μL of the sample in a 96-well microplate was measured at 734 nm with a plate reader (MULTISKAN GO, Thermo Fisher Scientific, Finland), analyzing the samples in triplicate. The results are expressed in mg equivalent of Trolox/g DW or in% radical inhibition ${\%}^{inhibición}=\frac{\begin{array}{c} \left(Abs.Inicial\right)-\left(Abs.Final\right) \end{array}}{Abs.Inicial}*100$.

#### 4.6.3. Antioxidant Capacity FRAP Method

The quantification of antioxidant capacity by FRAP (Ferric Reducing/Antioxidant Power) was carried out following the Olaya method [45]. The antioxidant capacity was determined by taking 20 μL of the methanolic extract and mixing with 230 μL of 2,4,6-tri(2-pyridyl)-s-triazine in a microplate. It was measured at 595 nm in a plate reader (MULTISKAN GO, Thermo Fisher Scientific, Finland), analyzing the sample in triplicate. The results were expressed in mg equivalent of trolox/g DW or in% radical inhibition ${\%}^{inhibición}=\frac{\begin{array}{c} \left(Abs.Inicial\right)-\left(Abs.Final\right) \end{array}}{Abs.Inicial}*100$.

### 4.7. Biological Activity

#### 4.7.1. α-Amylase Inhibition

The quantification of pancreatic α-amylase (Sigma Aldrich chemical A8273, St. Louis, Missouri, USA) was performed following the procedure described by MezaandValdés [46], modified for use for 96-well microplate. 450 μL of phenolic extract and 450 μL of α-amylase solution (0.5 mg/mL) are mixed in Eppendorf tubes, covered 25 °C/10 min. Subsequently, 450 μL of starch are added and cover at 25 °C/10 min. Finally, 300 μL of DNS (3,5-Dinitro-2-hydroxybenzoic acid) was added and subsequently heated in a water bath at 180 °C/5 min. Allow to cool to room temperature and placed in the 96-well microplate. Absorbance was measured at 540 nm in a plate reader (MULTISKAN GO).

#### 4.7.2. α-Glucosidase Inhibition

The α-glucosidase inhibition was performed following the procedure described by Ranilla et al. [4], modified for 96-well microplate. An amount of 50 μL of the methanolic extract, 50 μL of 0.1 M potassium phosphate buffer pH 6.9 and 100 μL of the α-glucosidase solution (1.0 U/mL) (Sigma Aldrich Chemical G3651, St. Louis, Missouri, USA) were mixed. They were covered at 25 °C/10 min in the microplate. Its absorbance at 405 nm was measured in a plate reader (MULTISKAN GO, Thermo Fisher Scientific, Finland). 50 μL of the 5 mM d-nitrophenyl-α-glucopyranoside solution is then added and placed at 25 °C/5 min, again its absorbance at 405 nm is recorded. Each experiment was performed in triplicate.

#### 4.7.3. Antihypertensive Activity

Inhibition of ACE (angiotensin-converting enzyme) was performed following the procedure described by Salazar Aranda, et al. [47], modified for 96-well microplate. 100 µL of the *N*-(3-(2-frapgglacryloy)) 5 × 10^−4^ M (Sigma Aldrich chemical F7131, St. Louis, Missouri, USA) solution, 125 µL of the methanolic extract, 15 µL of the TRIS-HCl buffer and 10 µL of ACE were mixed. The absorbance at 345 nm was measured for 5 min in a plate reader (MULTISKAN GO, Thermo Fisher Scientific, Finland). Analyzing the samples in triplicate.

### 4.8. Statistical Analysis

All the results obtained in this investigation were shown as the mean ± standard error. Data were evaluated using unidirectional ANOVA and changes between processing were evaluated by comparing means using the Tukey test. The level of statistical significance was considered at *p* < 0.05. Principal component analysis (PCA) with a self-scaling using the medium centering method and hierarchical clustering analysis (HCA) with centroid link grouping for the responses of the identified compounds were performed in order to characterize the effect of the treatments in the content of bioactive compounds, using XLSTAT 2014.05.03 (XLSTAT and Addinsoftare Registered Trademarks of Addinsoft. https://www.xlstat.com)

## 5. Conclusions

This study shows that the preliminary phytochemical analysis of the extracts demonstrated the presence of different groups of secondary metabolites (flavonoids, tannins and phenols). Therefore, in this paper, the vegetative material shows significant differences superior to the fresh vegetative material after applying the microwave drying and freeze-drying methods. On the other hand, the conventional method a thermal and/or enzymatic degradation of phenolic compounds was observed in the vegetative material. This effect was due to the leaves drying up at temperatures between 50 and 100 °C suffered an enzymatic and thermal degradation in the main factors of the reduction of phenolic compounds. Regarding the results obtained, it is concluded that the biological activity is affected by the type of drying with which the vegetative material. These drying methods significantly influence both positively and negatively the properties of the plant material, altering the amounts of phytochemicals present as Phenolic compounds and play an important role in enzyme inhibition and antioxidant capacity. Therefore, we can conclude that microwave drying, and freeze-drying have positive effects by increasing the percentages of inhibition of ACE, α-glucosidase, and α-amylase that contribute to the treatment of HTA and DMII. For all the herbs analyzed, drying was carried out mainly with decreasing rates. Initially, there was a relatively short phase with a higher moisture removal rate, with a high moisture content in the material. This phenomenon resulted from a greater ability to absorb microwave energy typical of material that contains more water. On the other hand, freeze-drying is used for the reliable preservation of a wide variety of heat-sensitive products and demands the highest standards of reliability and control. Unfortunately, the high porosity of dry materials has a negative effect on storage stability. Therefore, freeze-drying materials need to be stored in an airtight package. The freeze-drying process remains expensive, which restricts its application.

## Figures and Tables

**Table 1:** Phenolic compounds content in methanol extracts in Melissa, peppermint, thyme, and mint.

Melissa	Phenolic mg GAE/g DW						Flavonoids mg RE/g DW						Tannins mg CE/g DW					
Drying Method					F	*p*-Value *					F	*p*-Value *					F	*p*-Value *
**Fresh plant**	0.4452	**±**	0.0319	^a^	13.492	0.000	0.5308	**±**	0.0437	^a^	0.471	0.706	0.0005	**±**	0.000	^a^	457.046	0.000
**Microwave oven**	0.5491	**±**	0.0419	^b^			0.5520	**±**	0.0455	^a^			0.0016	**±**	0.001	^c^		
**Conventional**	0.4452	**±**	0.0053	^a^			0.5262	**±**	0.0216	^a^			0.0028	**±**	0.002	^d^		
**Freeze-drying**	0.5343	**±**	0.0408	^b^			0.5262	**±**	0.0433	^a^			0.0011	**±**	0.000	^b^		
**Peppermint**																		
**Fresh plant**	0.14030	**±**	0.00493	^a^	27.695	0.000	0.22140	**±**	0.02005	^a^	2.359	0.102	0.04905	**±**	0.00392	^b^	853.154	0.000
**Microwave oven**	0.14800	**±**	0.00649	^a^			0.25058	**±**	0.02269	^a^			0.00170	**±**	0.00011	^a^		
**Conventional**	0.12000	**±**	0.00526	^b^			0.23885	**±**	0.02163	^a^			0.00321	**±**	0.00017	^a^		
**Freeze-drying**	0.14400	**±**	0.00631	^a^			0.25006	**±**	0.02265	^a^			0.00196	**±**	0.00006	^a^		
**Thyme**																		
**Fresh plant**	0.24804	**±**	0.01869	^a^	15.407	0.000	0.93051	**±**	0.04695	^a^	125.032	0.000	0.01232	**±**	0.00124	^c^	501.007	0.000
**Microwave oven**	0.28973	**±**	0.02459	^b^			0.93051	**±**	0.04695	^a^			0.00072	**±**	0.00003	^a^		
**Conventional**	0.23492	**±**	0.01994	^a^			0.79758	**±**	0.04024	^b^			0.00064	**±**	0.00005	^a^		
**Freeze-drying**	0.31323	**±**	0.02658	^b^			0.53172	**±**	0.02683	^c^			0.00224	**±**	0.00004	^b^		
**Mint**																		
**Fresh plant**	0.10504	**±**	0.07925	^a^	0.054	0.983	0.77346	**±**	0.00773	^c^	200.684	0.000	0.00042	**±**	0.00042	^a^	4.988	0.010
**Microwave oven**	0.10162	**±**	0.10425	^a^			0.87538	**±**	0.00875	^a^			0.00004	**±**	0.00004	^ab^		
**Conventional**	0.09887	**±**	0.10144	^a^			0.81896	**±**	0.00819	^b^			0.00028	**±**	0.00028	^b^		
**Freeze-drying**	0.12084	**±**	0.12398	^a^			0.87356	**±**	0.00873	^a^			0.00016	**±**	0.00016	^a^		

mg GAE/g DW (mg Gallic acid equivalents/g dry weight), mg RE/g DW (routine equivalents/g dry weight), mg CE/g DW (mg catechin equivalents/g dry weight). The average value of 4 treatments, 3 repetitions. General multivariate linear model. * Comparison between means (Tukey α ≤ 0.05). Digits with equal letters in the same column are not statistically different. Different letters in the column indicate what are statistically different.

**Table 2:** Antioxidant capacity of secondary metabolites in methanol extracts in melissa, peppermint, thyme, and mint.

Melissa		DPPH %					ABTS %						FRAP %					
Drying Method					F	*p*-Value *					F	*p*-Value *					F	*p*-Value *
fresh plant	6.3	±	0.4	^d^	23.4	0.000	36.3	±	0.11	^d^	71.1	0.000	78.5	±	0.1	^b^	12.6	0.000
Microwave oven	41.8	**±**	0.7	^c^			39.9	**±**	0.4	^c^			89.5	**±**	0.3	^a^		
Conventional	73.5	**±**	0.2	^b^			48.3	**±**	0.1	^b^			9.3	**±**	0.9	^d^		
Freeze-drying	73.5	**±**	0.1	^a^			55.7	**±**	0.3	^a^			49.2	**±**	0.2	^c^		
**Peppermint**																		
fresh plant	40.4	**±**	0.8	^c^	37.2	0.000	64.8	**±**	0.7	^b^	17.8	0.000	56.2	**±**	0.1	^b^	10.9	0.000
Microwave oven	22.4	**±**	0.2	^d^			31.4	**±**	0.1	^d^			89.1	**±**	0.1	^a^		
Conventional	71.3	**±**	0.0	^b^			39.1	**±**	0.3	^c^			47.6	**±**	0.2	^d^		
Freeze-drying	77.5	**±**	0.3	^a^			78.8	**±**	0.2	^a^			54.0	**±**	0.5	^c^		
**Thyme**																		
fresh spices	6.64	**±**	0.4	^d^	36.6	0.000	34.3	**±**	0.4	^d^	20.2	0.019	57.5	**±**	0.4	^b^	48.7	0.000
Microwave oven	32.0	**±**	0.6	^c^			62.7	**±**	0.3	^a^			82.5	**±**	0.0	^a^		
Conventional	75.4	**±**	0.1	^a^			52.1	**±**	0.1	^c^			7.4	**±**	0.3	^d^		
Freeze-drying	74.4	**±**	0.1	^b^			56.2	**±**	0.1	^b^			53.6	**±**	0.0	^c^		
**Mint**																		
fresh plant	22.4	**±**	0.3	^d^	78.6	0.015	40.4	**±**	0.3	^d^	44.7	0.000	74.3	**±**	0.4	^b^	11.7	0.000
Microwave oven	39.5	**±**	0.2	^c^			53.3	**±**	0.4	^b^			88.1	**±**	0.2	^a^		
Conventional	76.4	**±**	0.2	^a^			49.4	**±**	0.3	^c^			42.7	**±**	0.2	^d^		
Freeze-drying	68.6	**±**	0.2	^b^			74.0	**±**	0.4	^a^			68.22	**±**	0.0	^c^		

The average value of 4 treatments, 3 repetitions. General multivariate linear model. * Comparison between means (Tukey α ≤ 0.05). Digits with equal letters in the same column are not statistically different. Different letters in the column indicate what are statistically different.

**Table 3:** Biological activity of secondary metabolites in methanol extracts in melissa, peppermint, thyme, and mint.

Melissa	α-Amylase %						α-Glucosidase %						ACE %					
Drying Method					F	*p*-Value *					F	*p*-Value *					F	*p*-Value *
Fresh plant	50.3	±	16.3	^ab^	10.1	0.000	70.5	±	1.3	^a^	40.5	0.000	7.73	±	0.6	^a^	43.1	0.000
Microwave oven	26.9	**±**	6.0	^b^			69.6	**±**	3.7	^a^			27.9	**±**	7.4	^b^		
Conventional	19.8	**±**	1.3	^b^			47.6	**±**	1.0	^b^			54.7	**±**	2.0	^c^		
Freeze-drying	84.1	**±**	3.6	^a^			31.0	**±**	3.9	^c^			72.6	**±**	4.4	^d^		
**Peppermint**																		
Freshplant	94.5	**±**	1.7	^a^	24.0	0.000	48.7	**±**	6.0	^ab^	17.2	0.000	54.6	**±**	2.2	^b^	41.4	0.000
Microwave oven	32.2	**±**	10.5	^c^			62.2	**±**	2.3	^a^			84.3	**±**	5.9	^c^		
Conventional	85.4	**±**	1.3	^ab^			26.5	**±**	1.0	^c^			29.5	**±**	2.0	^a^		
Freeze-drying	71.3	**±**	3.2	^b^			46.9	**±**	2.7	^b^			49.2	**±**	2.3	^b^		
**Thyme**																		
Fresh plant	24.0	**±**	8.2	^c^	23.1	0.000	50.7	**±**	3.2	^ab^	4.1	0.019	3.0	**±**	0.5	^a^	20.6	0.000
Microwave oven	57.8	**±**	7.2	^b^			63.0	**±**	2.8	^a^			20.9	**±**	2.8	^b^		
Conventional	91.3	**±**	1.5	^a^			46.3	**±**	3.1	^b^			8.5	**±**	2.2	^a^		
Freeze-drying	69.9	**±**	3.9	^ab^			56.6	**±**	4.6	^ab^			19.8	**±**	1.1	^b^		
**Mint**																		
Fresh plant	35.1	**±**	10.8	^ab^	4.4	0.015	39.1	**±**	3.7	^b^	14.1	0.000	49.3	**±**	0.9	^b^	180.8	0.000
Microwave oven	54.0	**±**	14.1	^ab^			64.1	**±**	3.9	^a^			71.9	**±**	1.4	^c^		
Conventional	24.5	**±**	7.0	^b^			58.5	**±**	1.7	^a^			14.4	**±**	0.9	^a^		
Freeze-drying	74.2	**±**	7.8	^a^			46.4	**±**	1.9	^b^			46.2	**±**	2.9	^b^		

The average value of 4 treatments, 3 repetitions. General multivariate linear model. * Comparison between means (Tukey α ≤ 0.05). Digits with equal letters in the same column are not statistically different. Different letters in the column indicate what are statistically different.

**Table 4:** Drying Parameters.

Drying Method	Plant Materials *	Moisture (%)	Drying Time (h)	Weight for Analysis (mg)		
				Phenolic Compounds	Antioxidant Compounds	Enzymatic Inhibition
**Fresh**	**Melissa**	**72.5**	-	200	200	200
	Peppermint	76.3	-	200	200	200
	Thyme	68.5	-	200	200	200
	Mint	76.9	-	200	200	200
**Microwave oven** **(30 GhZ/10 range)**	Melissa	5.9	0.16/10 range	25	25	25
	Peppermint	5.6	0.16/10 range	25	25	25
	Thyme	5.2	0.16/10 range	25	25	25
	Mint	5.6	0.16/10 range	25	25	25
**Conventional (58 °C)**	Melissa	6.9	25	25	25	25
	Peppermint	6.5	25	25	25	25
	Thyme	7.1	25	25	25	25
	Mint	6.4	25	25	25	25
**Freeze-drying** **(−50 °C/0.012 atm)**	Melissa	6.1	24	25	25	25
	Peppermint	5.3	24	25	25	25
	Thyme	5.9	24	25	25	25
	Mint	5.2	24	25	25	25

* Each sample adjusted the data with the dilution factor. Each data of the analysis was modified with respect to its percentage of humidity. The sample is presented per g of dry weight (DW).
